# Nucleolar Stress: hallmarks, sensing mechanism and diseases

**DOI:** 10.15698/cst2018.06.139

**Published:** 2018-05-10

**Authors:** Kai Yang, Jie Yang, Jing Yi

**Affiliations:** 1 Shanghai Key Laboratory for Tumor Microenvironment and Inflammation, Department of Biochemistry and Molecular Cell Biology, Shanghai Jiao Tong University School of Medicine, 280 South Chongqing Road, Shanghai, 200025, China.; 2 Shanghai Key Laboratory for Prevention and Treatment of Bone and Joint Diseases with Integrated Chinese-Western Medicine, Shanghai Institute of Traumatology and Orthopedics, Ruijin Hospital, Shanghai Jiao Tong University School of Medicine, 197 Ruijin 2nd Road, Shanghai, 200025, China.

**Keywords:** nucleolar stress, ribosome biogenesis, NPM1, translocation, p53, MDM2, ribosomal proteins

## Abstract

The nucleolus is a prominent subnuclear compartment, where ribosome biosynthesis takes place. Recently, the nucleolus has gained attention for its novel role in the regulation of cellular stress. Nucleolar stress is emerging as a new concept, which is characterized by diverse cellular insult-induced abnormalities in nucleolar structure and function, ultimately leading to activation of p53 or other stress signaling pathways and alterations in cell behavior. Despite a number of comprehensive reviews on this concept, straightforward and clear-cut way criteria for a nucleolar stress state, regarding the factors that elicit this state, the morphological and functional alterations as well as the rationale for p53 activation are still missing. Based on literature of the past two decades, we herein summarize the evolution of the concept and provide hallmarks of nucleolar stress. Along with updated information and thorough discussion of existing confusions in the field, we pay particular attention to the current understanding of the sensing mechanisms, i.e., how stress is integrated by p53. In addition, we propose our own emphasis regarding the role of nucleolar protein NPM1 in the hallmarks of nucleolar stress and sensing mechanisms. Finally, the links of nucleolar stress to human diseases are briefly and selectively introduced.

## INTRODUCTION

The nucleolus is a subnuclear compartment, which is primarily known for its role in ribosome biosynthesis. Within nucleoli, genes for ribosomal RNA (rDNA) are arranged in arrays of tandem repeats, precursors of ribosomal RNA (rRNA) are transcribed by the RNA polymerase I (Pol I) and processed, before the ribosomal proteins are incorporated and ribosomal subunits are assembled. However, during the past two decades, researchers have demonstrated that this is in fact an organelle having multiple complex functions. Several lines of evidence have revealed the most intriguing novel role of the nucleolus as a sensor for various cellular stresses, eventually leading to the concept of ‘nucleolar stress’. Numerous studies have listed triggers for nucleolar stress, characterized morphological and functional alterations, and dissected the molecules that induce activation of p53 signaling or other stress-responsive pathways. While these studies enriched our understanding of the general features of nucleolar stress, many questions, especially those regarding the sensing mechanism, remain unanswered. In this review we summarize the literature describing the evolution of the concept, focusing on the hallmarks and the sensing mechanisms for nucleolar stress. We will also discuss some key ambiguities in this field.

## THE NUCLEOLUS: A MULTIFUNCTIONAL ORGANELLE AND ITS ROLE IN CELLULAR STRESS

Starting in the 1990s, evidence has gradually accumulated that transcripts other than rRNAs can be produced and processed in the nucleolus 1. Indeed, a world of small nucleolar RNAs (snoRNAs) was discovered; these small RNAs are important for modifications of rRNA, tRNA and many small nuclear RNAs 2. In addition, the nucleolus was also found to functionally interact with Cajal bodies, other nuclear sub-compartments, to promote non-ribosomal RNA species maturation 3. Around the new millennium proteomics approaches were massively applied, which led to the identification of over 4500 nucleolar proteins in the nucleolus, of which only 30% have a clear relationship with ribosome biogenesis 4. This intriguingly showed that the nucleolus is involved in such diverse cellular events as signal recognition particle assembly, cell cycle regulation, DNA replication and repair, control of aging, response to viral infection, modulation of telomerase, and others 5,6. Overall, these clues indicate that the nucleolus is a multifunctional organelle.

In parallel, many researchers noticed that some nucleolus-enriched proteins are frequently shuttled between the nucleolus and nucleoplasm. Remarkably, a number of nucleolar proteins translocate to the nucleoplasm in response to various stress conditions 7,8,9,10,11,12,13. This phenomenon was initially observed when ribosome biogenesis was blocked by Actinomycin D (Act.D), a Pol I inhibitor 14, and soon thereafter was also found in cells exposed to cytotoxic agents 9,15, viral proteins 16, ultraviolet radiation 10,17, heat shock 15 and agents inducing DNA damage 18,19, apoptosis or senescence 20,21,22.

The links between the nucleolus and cellular stress were eventually proposed based on the findings that the nucleolus participates in regulating the abundance of the stress responsive protein p53 7,23,24,25,26,27,28. In summary, the notion that the nucleolus plays a role in regulating cellular stress states represents at least two aspects of the same idea: p53 activation by nucleolar proteins. One hypothesis emphasizes that the nucleolus is a sensor for cellular stresses, in which stress-induced nucleoplasmic translocation of nucleolar proteins, such as NPM1 29,30,31 and GLTSCR2 32 initiates p53 activation. The other hypothesis proposes the engagement of ribosomal proteins (RPs) mainly RPL5 33, RPL11 34 and RPL23 35, or nucleolus-resident proteins (e.g. ARF 36 among others) in p53 interaction with its negative regulator MDM2, but placing less emphasis on their translocation.

## THE DEFINITION AND EVOLUTION OF THE CONCEPT OF NUCLEOLAR STRESS

**Figure 1:**
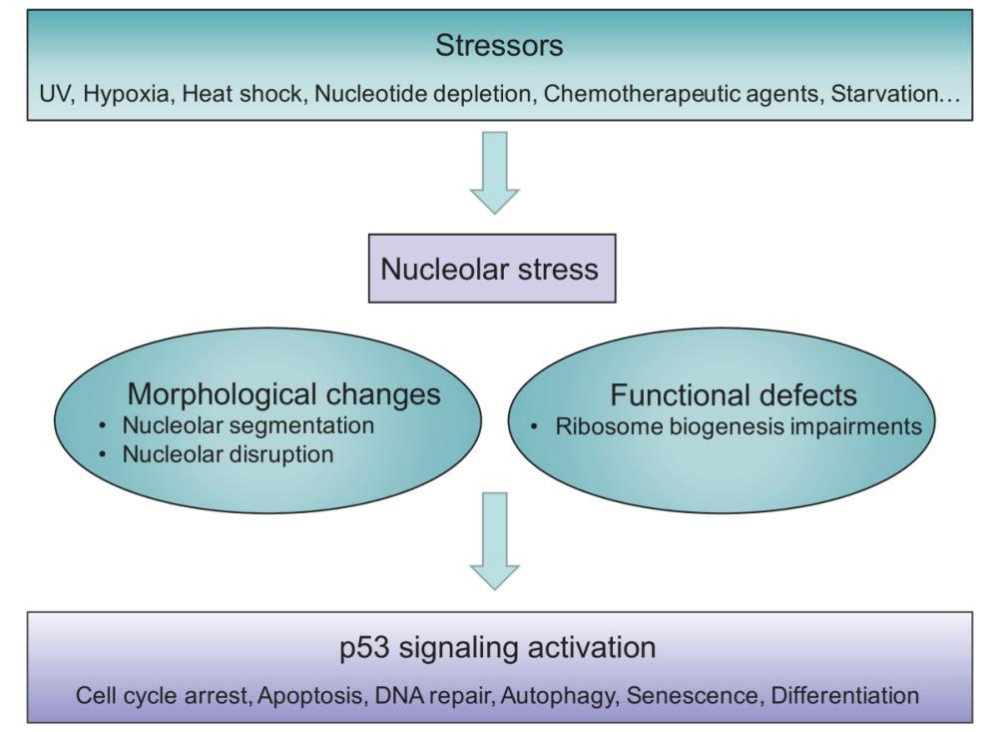
FIGURE 1: Schematic illustration of nucleolar stress. Various stressors induce nucleolar stress, accompanied by morphological changes and functional defects, ultimately resulting in activation of p53 signaling pathway and altered cell behavior.

Although the term ‘nucleolar stress’ is increasingly used, the exact description varies and (still) evolves, thus a precise definition has not yet been universally approved. The term was originally referred to the stressful events that impair the homeostasis of ribosome biogenesis and activate the cellular stress response. Therefore, nucleolar stress has also been referred to as ‘ribosomal stress’ or ‘ribotoxic stress’ 37,38,39,40, as typical inducers are the Pol I inhibitor Act.D 14, or aberrant expression of nucleolar proteins 25, which also impair ribosomal function. In general, nucleolar stress is now used to describe various stressor-induced impairments in nucleolar morphology and function that ultimately lead to disturbances in cell homeostasis through activation of p53 or other stress signaling (**Figure 1**).

The idea that cell cycle progression may depend on some aspect of ribosome biogenesis was first implied in early studies on the cell cycle 41. In higher eukaryotes, Volarevic proved that deletion of RPS6 in the liver of adult mice abolished 40S ribosome biogenesis and inhibited cell proliferation following partial hepatectomy 42. Identifying the correlation of interference of the nucleolar protein Bop1 and p53-dependent cell cycle arrest, Pestov et al. proposed that perturbation in ribosome biogenesis may cause nucleolar stress, leading to cell cycle arrest in a p53-dependent manner 25. Indeed, this model of nucleolar stress, probably the first of its kind, is consistent with many observations under diverse p53-activating stressors 28,29,43.

The pioneering work conducted by Rubbi and Milner significantly solidified the notion of nucleolar stress 29. They aimed at resolving the puzzle of how signals in a large variety of cellular stress situations can be integrated by a single molecule, namely p53. A common phenomenon in all p53-inducing stresses is nucleolar disruption. Based on a comparative meta-analysis of diverse stimuli that activate p53 signaling and induce nucleolar alteration, they hypothesized that the impairment of nucleolar function might stabilize p53. In fact, activation of p53 is induced by a wide range of cellular stresses, aside from the Pol I inhibitor Act.D, which all cause disruption of nucleolar organization. The translocation of nucleophosmin (NPM1, or B23), an abundant nucleolar protein that is the most frequently reported to move to the nucleoplasm and cytoplasm upon various cellular insults was set as the criterion for nucleolus disruption. Rubbi and Milner demonstrated that NPM1 translocation, or nucleolus disruption following micropore UV irradiation over the nucleoli occurs prior to and independent of p53 induction. Alternatively, p53 response can be induced by interfering with nucleolar function using an antibody against the nucleolar protein UBF (upstream binding factor) in the absence of any genotoxic insult. Therefore, the model they proposed was the only one that could provide a unifying and coherent explanation for the action of all known p53-stabilizing agents.

## THE HALLMARKS OF NUCLEOLAR STRESS

Following the principle of cellular events in response to stress conditions, we describe the following elements as the hallmarks of nucleolar stress.

### Ribosome biogenesis insults and a wide range of stimuli as stressors 

Ribosome biogenesis comprises multiple steps accomplished in three distinct subnucleolar components, from Pol I transcription initiation to pre-rRNA processing and ribosomal assembly. Any error that causes disturbance in ribosome biogenesis will lead to nucleolar stress.

In fact, deletion or aberrant expression of a number of ribosomal proteins induce p53 stabilization and activation via disruption of ribosome biogenesis: Pestov *et al*. found that perturbation of the nucleolar protein Bop1 activity could induce ribosome biogenesis impairment, followed by a p53-dependent cell cycle arrest 25. Genetic inactivation of TIF-1A, a basal transcription initiation factor for Pol I, leads to nucleolar disruption, cell cycle arrest and p53-mediated apoptosis 44. Depletion of importin 7 (IPO7) or exportin 1 (XPO1) proteins impairs ribosome biogenesis and also initiates p53-dependent cell cycle arrest 45. Microinjection of specific monoclonal antibodies against transcription factor UBF inhibits rRNA transcription and leads to p53 stabilization 29. Overall, systematic screening analysis revealed an extensive connection of p53 stabilization with nucleolar disruption induced by ribosomal protein depletion 46.

The chemotherapeutic agent Act.D is the mostly used nucleolar stress inducer. It may inhibit three individual RNA polymerases at different concentrations 47. It is believed that Act.D can induce DNA damage and inhibit general transcription at high concentrations, such as 430 nM, but selectively inhibits Pol I and induces ribosomal stress at low dose like 5 nM 34.

Strikingly, as summarized by Rubbi and Milner, stressful conditions that can induce p53 activation can all induce nucleolar stress: these include UV light, hypoxia, heat shock, nucleotide depletion and various chemotherapeutic agents 29. These stimuli were confirmed to simultaneously induce nucleolar stress and p53 activation by subsequent studies 48,49. The rationale that stressors of diverse nature can all induce nucleolar stress has not been adequately discussed, thus has remained unknown for a long time. Recently we found that common cellular insults that are able to induce p53 activation can also induce the translocation of NPM1, a hallmark of nucleolar stress, in a reactive oxygen species (ROS)-dependent manner. Moreover, our study added nutrient starvation and direct exposure to hydrogen peroxide (H_2_O_2_) to the growing list of nucleolar stress inducers 31.

**Figure 2:**
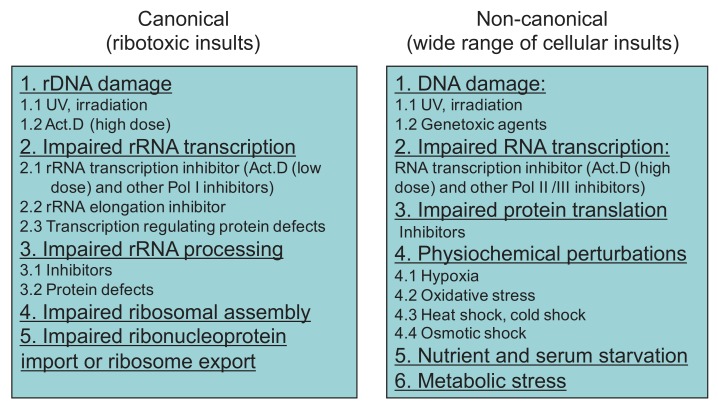
FIGURE 2: Stressors eliciting nucleolar stress. Two categories of nucleolar stress inducers are direct ribotoxic insults and a wide range of cellular insults.

In summary, the reported factors that induce nucleolar stress can be classified into two categories: canonical and non-canonical. The former points to those affecting homeostasis of ribosome biogenesis, whereas the latter includes a wide range of general cellular insults (**Figure 2**).

### Nucleoplasmic translocation of nucleolar proteins

Unlike membrane-limited organelles, there is no structural barrier between the nucleolus and the surrounding nucleoplasm. As a consequence, any soluble molecule can potentially traffic in and out of the nucleolus in a relatively free manner. This shuttling might occur at basal levels under non-stressful ‘resting’ conditions, but is significantly increased under various stress conditions. Nucleolar stress causes a lot of nucleolar molecules to redistribute in the nucleus, or in other words, to be released from the nucleolus to the nucleoplasm. This translocation or redistribution is thus considered as an indicator of nucleolar stress.

#### NPM1

NPM1 (also known as B23, nucleophosmin, numatrin or NO38) is the most abundant protein in the nucleolus 50,51 and under diverse scenarios can dynamically shuttle both within nucleoli and between the nucleolus and the nucleoplasm or the cytoplasm 52,53,54. The known functions of this protein include the interaction with a plethora of macromolecules, for instance, Rb in the nucleus 55 and BAX in the cytoplasm 56, and chaperoning activity protecting proteins from aggregation in the crowded nucleolar environment 57,58. At exit of mitosis, NPM1, among other ribosomal processing proteins, undergoes bidirectional traffic between incipient nucleoli and perinucleolar bodies, which may contribute to nucleolar assembly in early G1 phase 59. NPM1 is also responsible for the nuclear export of ribosomal protein L5 53.

Studies on NPM1 nucleoplasmic translocation are mostly based on contributions made by Busch, Chan and Yung 52. Although the concept of nucleolar stress had not been proposed by then, the conditions under which they found NPM1 nucleoplasmic translocation belonged to general cellular stress or typical ribosomal stress. With immunofluorescence technology, they first found that upon 48 hours serum-free medium starvation, NPM1 was diminished in nucleoli and appeared in the nucleoplasm, whereas refeeding of serum-containing medium relocated NPM1 protein to nucleolus 60, indicating a reversible nucleoplasmic translocation capability of NPM1. They also noticed that ribosomal transcription inhibitors, such as Act.D, were all able to induce NPM1 nucleoplasmic translocation 52.

Furthermore, a wide range of anticancer agents aside from specific inhibitors also induce NPM1 nucleoplasmic translocation, including the inosine-5'-monophosphate (IMP) dehydrogenase inhibitor tiazofurin 61, DNA topoisomerase II (topo II) inhibitors doxorubicin and daunomycin 8,62, topo I inhibitors mitomycin C and camptothecin 63,64, phosphatidylinositol kinase inhibitor toyocamycin 65 and JAK/STAT3 inhibitor cucurbitacin B 21. Even an iron chelator deferoxamine which showed anti-proliferation effects 66, UV radiation 30, viral infection 30, hypoxia and oxidative stress (H_2_O_2_) 31 all lead to nucleoplasmic translocation of NPM1.

Among the observations of nucleoplasmic translocation of NPM1, a great part described the association of this event with p53 signaling activation. Using the anti-cancer drug daunomycin, Chan *et al*. found a relationship between NPM1-translocation and apoptosis 62. Then, Rubbi *et al*. elegantly proved a relationship between NPM1 translocation and p53 activation using different doses of UV irradiation in nucleolar areas and different anti-cancer drugs 29. Furthermore, Kurki *et al*. found that UV irradiation-induced p53 activation was dependent on NPM1 interaction with HDM2, suggesting that NPM1 activates p53 in a regulated fashion 30. Recently, we uncovered a redox mechanism of NPM1 for sensing nucleolar stress that causes p53 accumulation and activation 31.

Therefore, as a most frequent event, NPM1 translocation should be regarded as a conspicuous hallmark of nucleolar stress.

#### Other nucleolar proteins

The following nucleolar proteins exhibit nucleoplasmic translocation under particular types of nucleolar stresses. However, their translocations are not yet explored as universally under many stress conditions as NPM1.

Nucleolin, alias C23, a DNA and RNA binding protein, is essential for pre-RNA transcription, folding, processing and assembly 67. Cyclin-dependent kinase inhibitor roscovitine induced both nucleolin translocation and nuclear accumulation of p53 7. Nucleostemin that functions in pre-RNA processing was also translocated to the nucleoplasm under doxorubicin and Act.D treatments in neonatal rat cardiomyocytes, which occurred concurrently with p53 accumulation 20.

PICT1, also called glioma tumor-suppressor candidate region gene 2 (GLTSCR2), a candidate tumor suppressor, is translocated to the nucleoplasm in response to hypoxia or Act.D treatment and enhanced p53 stability through ARF-independent direct physical interaction with p53 32.

### Morphological descriptions for nucleolar stress

According to the classical ‘tripartite’ model, the three main events for ribosome biogenesis, i.e., pre-rRNA transcription, processing, and ribosomal subunit assembly, are reflected in three distinct subnucleolar compartments named the fibrillar center (FC), the dense fibrillar component (DFC), and the granular component (GC). It is generally accepted that pre-rRNA is transcribed from rDNA in the FC or at the border between the FC and DFC. FCs are enriched in components of the RNA Pol I machinery, such as UBF, whereas the DFC harbors pre-rRNA processing factors, such as the snoRNAs and snoRNP proteins, fibrillarin and Nop58. Both the FC and the DFC are surrounded by the GC, where pre-ribosome subunit assembly takes place (reviewed in 5,68). The morphology and size of nucleoli are linked to nucleolar activity, which are inevitably altered under stress conditions, showing a variety of reorganization.

The widely used descriptions of morphological alterations in nucleolar stress are based on immunostaining using fluorescence-labeled antibodies against known markers of the nucleolus, such as NPM1, fibrillarin, and UBF, that visualizes their redistribution under nucleolar stress conditions. Typically, under Act.D treatment, these nucleolar marker proteins aggregate in different regions, migrate towards the nucleolar periphery, or even distribute to the nucleoplasm, finally forming distinct staining structures, the nucleolar caps, and spots or foci that spread in the nucleoplasm. Nucleolar caps are shaped around the nucleolar remnant; they can be also formed by nucleoplasmic proteins (mostly RNA-binding proteins) or the Cajal body marker, coilin 69.

These kinds of morphological alterations have been designated as ‘nucleolar segregation’ or ‘nucleolar disintegration’ to reflect a state of loss of nucleolar integrity. As reviewed by Boulon *et al*., segregation is characterized by the condensation and subsequent separation of the FC and GC, together with the formation of nucleolar caps 49. Nucleolar segregation is thought to be different from nucleolar fragmentation which occurs following inhibition of either RNA Pol II (but not I) or protein kinases 7,70 and leads to unraveling of the FC. Viral infections can also cause specific changes in nucleolar morphology, such as an increase in nucleolar size 71.

Nucleolar segregation has emerged as an indicator of nucleolar stress induced by, in particular, agents that cause rDNA damage and rRNA transcription impairment 72,73,74. For instance, chemotherapeutic agents that inhibit rRNA transcription and early processing steps, but not late processing steps, lead to the loss of nucleolar integrity, which is marked by NPM1 translocation to the nucleoplasm 75. Meanwhile, after chemotherapeutic agent treatment that mostly inhibit early and late rRNA processing steps, fibrillarin and the ribosomal biogenesis factor pescadillo are translocated into distinct morphological subnuclear structures, namely nuclear spots and nucleolar caps structures or even form ‘necklace’ structures (especially for fibrillarin) 75. Interestingly, it was noticed that the nucleolar integrity was maintained for drugs without inhibitory activity on ribosome biogenesis 76.

**Figure 3:**
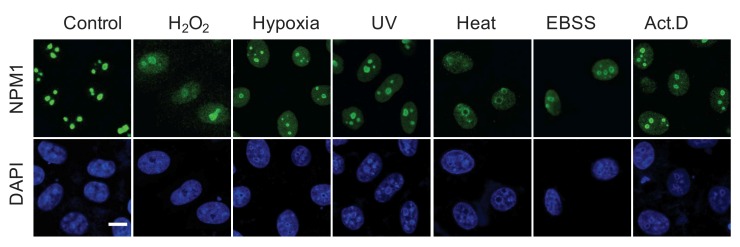
FIGURE 3: NPM1 translocation under nucleolar stress. Representative images of NPM1 translocation in HeLa cells under various nucleolar stresses, including H_2_O_2_ (500 (M, 30 min), hypoxia (1% O_2_, 1 h), UV irradiation (100 J m^-2^), heat-shock (42°C, 30 min), EBSS starvation (6 h) and Act.D (8 nM, 1 h), examined by immunofluorescence with anti-NPM1 antibody. Bar, 5 (m. Image from ref 31

A more frequently used term for morphological alteration is ‘nucleolar disruption‘ that was initiated by Rubbi and Milner, meaning the dispersion of the nucleolar structure 29. Notably, this morphological description is characterized by the release of nucleolar proteins, in particular, NPM1, to the nucleoplasm, being seen as a homogenous staining in nucleoplasmic area or even whole nuclear area 29,31,52,60 (**Figure 3**). This characteristic alteration may simultaneously occur with the formation of nucleolar caps and foci, but is frequently used to independently indicate nucleolar stress.

Electron microscopic observations showed a segregation of nucleolar components when cells were exposed to the antitumoral drug VM26, a specific inhibitor of topoisomerase II 77. Drug treatment caused double-strand breaks in the tandem repeat rDNA genes, leading to rDNA fragmentation, which might explain the morphology with a segregation of nucleolar components. Double immuno-gold labeling demonstrated redistribution of the nucleolar or nuclear proteins during nucleolar stress 78. However, the immuno-electron microscopic characteristics of the nucleoli are technically challengeable and less quantitative compared to immunofluorescence under light microscope, thus are not widely applicable.

Interestingly, nucleolar atrophy is observed in neurons of patient brain autopsies 79 and pharmacological mouse models for some neurodegenerative disorders like Parkinson’s disease and Alzheimer’s disease 80,81, indicating that the size of the nucleolus can change under long-term stress. Of note, changes in nucleolar size and shape in cultured cells can be briefly observed by simple phase contract microscope 82. We list the general morphological characteristics of nucleolar stress in **Table 1**.

**Table 1:** TABLE 1. Morphology upon nucleolar stress.

**Microscopy**	**Observations**	**Related nuclear proteins**
Phase contrast	Reduced nucleolar size	
Immunofluorescence	1. Nucleolar caps (in nucleolus)	Fibrillarin, UBF
	2. Necklaces, rings (in nucleolus)	Fibrillarin, UBF, NPM1
	3. Spots, foci (out of nucleolus)	Fibrillarin, UBF, NPM1
	4. Disruption	NPM1
Electron microscopy (EM)	Reduced nucleolar size, segregation	
Immuno-EM	Segregation	Fibrillarin, UBF, NPM1

### Impaired rRNA transcription and processing

In mammalian cells, precursor ribosomal RNA (47S pre-rRNA) is transcribed by Pol I, then processed to 45S, 41S, 36S, and 32S rRNA intermediate precursors and finally matured 18S, 5.8S and 28S rRNA 83. To curtail the role of rRNA maturation in nucleolar stress and to identify specific processing steps, which might be impaired under the respective stress condition, a number of methodological approaches are available: Inhibition of rRNA transcription can easily be confirmed by reduced amount of 47S pre-rRNA precursor. This could happen under Pol I-targeted chemotherapeutic drugs treatment 75, depletion of essential factors in Pol I complex (like TIF-IA and UBF), or specific Pol I component inhibitors (like Act.D) 47. Inhibition of rRNA processing usually results in accumulated precursor levels, reduced amounts of products or both. If an early processing stage is impaired, it will result in reduced 41S, 36S and 32S rRNA products 75; if a late stage is impaired, there will be accumulated 32S rRNA or reduced 28S rRNA 25,84. The relative ratio changes of precursor and product (like 32S/28S) is widely accepted as a more accurate measurement of the processing 85,86. Eventually, by analyzing the variation of the amount of precursors and products after treatments, one can figure out which steps in ribosome biogenesis are impaired.

Ethidium bromide (EtBr) staining and reversed-transcriptional PCR (RT-PCR) can be used as classical nucleic acid detecting methods for analyzing individual rRNA products. However, to get a more accurate result, radioactive probes for specific rRNAs and Northern hybridization analysis of precursors are widely applied 87. For instance, with ^32^P or ^3^H labeling in culture medium, the major rRNA precursors can be visualized 75, and 32S precursor can be detected using radioactive ITS2-specific probes 88.

### Activation of p53 signaling

The p53 tumor suppressor protein is considered as an integration point in response to various cellular stresses 89,90. The activation of p53 can promote transcription of p21 leading to G1/S growth arrest 91, of 14-3-3 sigma inducing G2/M arrest 92, or of Bax inducing apoptosis 93. It can also induce other factors involved in autophagy, DNA repair and metabolism 94.

The major negative regulator of p53 is the E3 ubiquitin ligase MDM2 (murine double minute 2, HDM2 in human). Mechanistically, MDM2 interacts with p53 via its C-terminal RING finger domain, promoting p53 ubiquitination and degradation by the 26S proteasome 95,96. Therefore, p53 stabilization and activation in response to various stresses rely on a disruption this interaction between p53 and MDM2/HDM2. Simple readouts for p53 activation under nucleolar stress conditions are an increased p53 protein levels (stabilization or accumulation following blockage of ubiquitin-proteasomal degradation), reduced p53 binding to MDM2/HDM2, increased p53 mRNA levels under a long-lasting stress, elevated mRNA levels of p53 target genes, typically *CDKN1A* (p21) and *BAX*, and corresponding cell phenotypes such as cell cycle arrest, autophagy, DNA repair, senescence, or apoptosis 89.

### Involvement of p53-independent stress signaling

In p53-/- or p53 inactivated cell lines, nucleolar stress can usually still invoke cell cycle arrest or apoptosis, implying that there is a stress response that is mediated by signaling pathways other than p53 97.

#### Ribosomal proteins (RPs) regulating transcription factors (TFs)

The major non-p53 TFs that respond to ribosomal stress are c-Myc, E2Fs and SP1 97,98,99,100. Their downregulation or decreased transcriptional activities by RPs mediate cellular stress responses via altered transcription of target genes. Measurement of mRNA and/or protein levels of these TFs and their target genes, and analysis of TF binding with the target DNA, may indicate the involvement of these signaling pathways.

The oncoprotein c-Myc positively controls cell growth and proliferation 101 and serves as a direct regulator of ribosome biogenesis; many products of its transcriptional target genes are involved in ribosome biogenesis 102. As a feedback mechanism, RPL5 and RPL11 are two critical negative regulators of c-Myc expression during ribosomal biogenesis; they form a complex with c-Myc mRNA and recruit microRNAs to repress c-Myc expression thus inhibiting the transcriptional activity of c-Myc 100. RPS14 may also function as a negative regulator of c-Myc 103. Consistently, upon nucleolar stress, as ribosome-free RPs, these proteins can lead to inhibition of cell proliferation through suppression of c-Myc and its target gene expression 102.

E2F-1 is a member of the E2Fs family of transcription factors; the expression of their target genes are important both for cell proliferation and apoptosis 104. Independent of its regulatory control of p53, MDM2 prolongs the half-life of E2F-1 99. Under impaired rRNA biosynthesis, free RPL11 binds to MDM2 causing E2F-1 degradation, which is associated with the inhibition of cell proliferation 105.

Recently, RPL3 has been found as a pro-apoptotic factor under nucleolar stress induced by 5-fluorouracil in colon cancer cells devoid of p53. RPL3 in ribosome-free form, negatively regulates cystathionine-β-synthase (CBS) expression at the transcriptional level through inhibition of Sp1 binding to the *CBS* gene 98. In addition, RPL3 can mediate p53-independent p21 upregulation, which requires the specific interaction between RPL3 and Sp1. Depending on its intracellular levels, p21 can either induce G_1_/S arrest of the cell cycle or mitochondria-mediated apoptosis 106.

#### RPs regulating non-TF proteins

RPL3 can not only negatively regulate CBS expression at the transcriptional level, but also trigger CBS translocation into mitochondria. Consequently, apoptosis is induced through the mitochondrial apoptotic cell death pathway 98.

#### Nucleolar proteins regulating TFs and non-TF proteins

There are several nucleolar proteins that bypass p53 and directly promote cell cycle arrest or apoptosis. These p53-independent regulators of apoptosis mainly include NPM1, PPAN, ARF and NuMA 107,108. Both NPM1 and ARF are well-known for their roles in p53 signaling, however, several reports have demonstrated their involvement in p53-independent signaling 109. In these cases, translocation of the nucleolar proteins and their interactions with the corresponding proteins may be analyzed. Interestingly, many RP or other nucleolar protein-mediated p53-independent stress responses require NPM1. In fact, NPM1 alone also interacts with apoptotic proteins. In conditions of nucleolar stress, NPM1 is transcriptionally induced and relocalizes from the nucleolus to the cytoplasm where it complexes with BAX, a crucial effector of the mitochondrial apoptosis pathway. Of note, cytosolic NPM1-BAX interaction has also been associated with cell resistance to death stimuli 109, therefore, the cellular response this direct interaction of NPM1 with apoptosis regulators does not necessarily result in cell death.

The Wnt target Peter Pan (PPAN) localizes to mitochondria in addition to its nucleolar localization and inhibits the mitochondrial apoptosis pathway in a p53-independent manner. Its role as an anti-apoptotic factor is indicated by the fact that knockdown of PPAN induces BAX stabilization, mitochondrial membrane depolarization and cytochrome c release. Staurosporine or Act.D-induced nucleolar stress and apoptosis disrupt nucleolar PPAN localization and induce its accumulation in the cytoplasm, which might be associated with impairment in its anti-apoptotic function 110.

Recently, the nuclear mitotic apparatus protein NuMA that locates in nucleoli in the interphase, has been demonstrated to be redistributed upon Act.D or doxorubicin- induced nucleolar stress. NuMA co-immunoprecipitates with Pol I, with RPL26 and RPL24, and with components of an ATP-dependent chromatin remodeling complex associated with rDNA transcription. Downregulation of NuMA expression triggers nucleolar stress, as shown by decreased nascent pre-rRNA synthesis, fibrillarin perinucleolar cap formation and upregulation of p27kip1, but not p53 108.

Several studies reported that ARF binds and antagonizes the transcriptional activities of c-Myc and E2F-1, halting cell cycle progression in absence of p53 111. In addition to the regulation of the TFs, ARF controls proliferation by limiting nucleolar localization of the RNA helicase DDX5, which ultimately increases ribosome output 112.

## THE MECHANISMS UNDERLYING NUCLEOLAR STRESS SENSING AND INTEGRATION TO p53 SIGNALING

Cumulative findings that any impairment in ribosome biogenesis by various insults can lead to p53 stabilization and activation, has led to the hypothesis that a low p53 level under non-stress condition relies on normal homeostasis of ribosome biogenesis in the nucleolus. This default state is ensured by the intactness of the ribosome biogenesis procedures and nucleolar structure. The evidence supporting this default model is robust, as p53 activation in nucleolar stress can be induced by the aberrant expression of those nucleolar proteins that are indispensable for ribosome biogenesis, or by various stimuli.

This notion then brought up an outstanding question how the errors or hurdles within the nucleolus signal to p53. Of note, since p53 is mainly controlled by its negative regulator MDM2/HDM2, one should specifically ask, how nucleolar stress interrupts MDM2-p53 association. We here summarize several aspects of studies addressing this question 30,48,97,113, including some frequent confusions or ambiguities.

### From nucleolar stress to MDM2/HDM2-p53 

#### The role of ribosomal proteins 

In response to nucleolar stress, several RPs bind to MDM2 and block MDM2-mediated p53 ubiquitination and degradation, resulting in p53 stabilization and activation. After cells are exposed to low doses of Act.D, serum starvation or other insults, there is an increased binding of RPL5, RPL11 and RPL23 to MDM2 33,34,114,115. RPL5, RPL11, and RPL23 all bind to the central acidic region of MDM2, but importantly, each of them requires specific sequences to interact 43,116,117. Therefore, the RP-MDM2-p53 signaling pathway has been proposed and believed to constitute a surveillance network monitoring the integrity of ribosomal biogenesis 113.

If an increased binding of free RPs with MDM2 is a prerequisite for this monitoring or sensing, the questions arises where these increased free RPs come from under nucleolar stress conditions, and in which subnuclear compartment they interact with MDM2, given that RPs mostly reside in the nucleolus and the cytoplasm, whereas MDM2 often stays in the nucleoplasm. However, these critical points have not been paid adequate attention to, and thus remain unclear. To our knowledge, first of all, few publications addressed the issue of subnuclear localization or fractionation of RPs and MDM2/HDM2 before and after stressor exposure. And secondly, the reported findings were controversial and highly context-dependent:

The early work by Lohrum *et al*. 114 showed that in unstressed U2OS cells under ectopic expression, (RP)L11 and HDM2 displayed complex localization. When expressed alone, L11 was predominantly nucleolar and HDM2 confined to the nucleoplasm, but 24 hours after co-transfection, L11 and HDM2 were both localized to the nucleoplasm when the L11:HDM2 ratio was low (2:1); or both localized to the nucleolus when L11 levels were higher (4:1). However, again under ectopic expression, in response to nucleolar stress induced by low levels of Act.D, both L11 and HDM2 co-localized with endogenous NPM1 to discrete subnuclear bodies, and, after longer treatment, both were co-localized with NPM1 in the nucleoplasm. Apparently, one is hardly able to draw conclusions from these observations that L11 is released from the nucleolus to the nucleoplasm upon stress.

Bursac *et al*. reported differential events in 2012 74. Using cell fractionation to purify the nucleolus extract, they found that endogenous nucleolar L5 and L11 were not reduced upon Act.D treatment, and using YFP-L11 transfection and fibrillarin immunostaining, they found that L11 was not translocated to the nucleoplasm upon Act.D treatment. The authors claimed that whereas several other newly synthesized ribosomal proteins are degraded by the proteasome upon Act.D treatment, L5 and L11 accumulate in the ribosome-free fraction where they bind to MDM2. Furthermore, the endogenous, newly synthesized L5 and L11 continued to be imported into nucleoli even after nucleolar disruption and co-localized with MDM2, p53, and PML. Therefore, in contrast to findings by others, their results suggest that the disrupted nucleoli may provide a platform for L5- and L11-dependent p53 activation.

Watkins and Thomas labs both pointed out that 5S RNA complexes with L5 and L11, and functions either in pre-60S assembly, or inhibiting MDM2 induced p53 degradation 118,119.

Recently, using immunostaining, Kayama *et al*. demonstrated a nucleoplasmic translocation of FLAG-tagged RPL11 in HCT 116 cells upon low dosed Act.D treatment 120.

In our experiments, immunostaining of RPL11 in U2OS cells showed a predominant location in the cytoplasm and no redistribution to the nucleoli or nucleoplasm was detectable upon Act.D-induced nucleolar stress (unpublished data).

A number of review articles stated or implied that the increased levels of ribosome-free RPs may originate from disrupted nucleoli 97,113. To our knowledge, this notion lacks direct evidence, and thus remains speculative, unless free endogenous RP translocation from the nucleolus is detected under specific or general stress conditions.

An increased RPs-MDM2 interaction could follow diverse cellular disturbances, such as global translation inhibition 121, or the breakdown of ribosomal polysomes in the cytoplasm 33,43,115. One can predict that there would be an accumulation of free RPs in the cells under these circumstances. However, in order to validate the postulation that excessive and ‘wandering’ free RPs find their way toward the nucleoplasm and there interact with MDM2, more detailed analysis based on subcellular fractionation and localization approaches are required. Taken together, it is worth further investigating how free RPs sense the nucleolar stress and then transmit signals to p53.

#### The role of ARF

ARF (alternative reading frame protein, p19 in mouse, p14 in humans) induces p53 activation in response to certain types of DNA damage 122 or several ontogenetic stresses 123,124,125, and is therefore categorized as a tumor suppressor. ARF is considered to localize to nucleoli of non-stressed cells 126. As a basic nucleolar protein rich in arginine residues, it binds to multiple ribosomal proteins, and participates in 47S rRNA transcription and 32S processing events 127. Although several studies revealed that ARF was released to nucleoplasm under nucleolar stress and targeted the central acidic domain of MDM2 to inhibit p53 degradation 128,129, some other reports implied that a resident nucleoplasmic fraction of total ARF is involved in the interaction with MDM2 under stress conditions 128,130. Moreover, some studies indicated that ARF is dispensable for nucleolar stress-induced p53 accumulation 131,132.

In our study 31, we found that endogenous or exogenous ARF was similarly distributed both in the nucleolus and nucleoplasm of U2OS cells; the nucleolar ARF did not move out following Act.D treatment and, in contrast, the nucleolus/nucleoplasm ratio of ARF was slightly increased, while NPM1 was redistributed to the nucleoplasm under the same condition.

Therefore, the role of ARF in nucleolar stress-induced p53 activation may be cell type-dependent and/or context-dependent. In addition, the subnuclear compartment where ARF interacts with MDM2 still needs to be determined.

#### The role of translocated nucleolar proteins

As outlined above, NPM1 can interact directly with p53 via its C-terminal domain 17 and also binds to HDM2, competing with p53 and blocking HDM2-mediated p53 ubiquitination 30. The wide range of stress conditions which provoke NPM1 translocation 16,31,133,134 might explain how p53 can integrate various stimuli. In our own work, we aimed at answering the question, if the translocated, nucleoplasmic fraction of NPM1 was the sole trigger for p53 activation. Therefore, we constructed an NPM1 mutant, unable to move upon Act.D treatment. In contrast to wild-type NPM1, this mutant was unable to disrupt the p53-HDM2 interaction, and thus greatly compromised the activation of p53 31. Interestingly, compared with the mock DNA, overexpression of the p53 activator ARF in U2OS cells with either normal or silenced NPM1 led to an increased accumulation of p53 under non-stress condition. However, after cells were exposed to Act.D, overexpression of ARF alone did not produce further accumulation of p53 in NPM1 knockdown cells. An enhanced p53 accumulation was observed when wild-type NPM1 was restored, but reintroducing the unmovable mutant NPM1 could not cause an increase of p53. A co-immunoprecipitation assay showed that the disruption of the HDM2-p53 interaction occurred in the cells bearing the wild-type but not mutant NPM1, whereas amounts of ARF bond to HDM2 appeared similar in both cell groups. These data suggest that the nucleoplasmic fraction of ARF alone is able to induce p53 accumulation under basal conditions. However, further p53 accumulation under stress conditions is determined by the presence of the nucleoplasmic NPM1, independent of ARF.

We also found that the p53-HDM2 interaction was prevented only by the wild-type but not the mutant NPM1 that lost nucleoplasmic translocation, whereas RPL23 remained bound to HDM2 in both samples. This means that, although RPL23 is required for p53 stabilization under stress, only NPM1 translocation to the nucleoplasm dictates the final outcome of p53 accumulation through NPM1’s binding with HDM2 and thus the disruption of the HDM2-p53 interaction. Collectively, the binding of ribosomal proteins or ARF with HDM2, which had been thought to be sufficient for p53 stabilization 33,115,135,136, is actually insufficient when NPM1 stays within the nucleoli. These results highlight that nucleoplasmic translocation of NPM1 is a prerequisite for stress-induced activation of p53.

### From nucleolar stress to NPM1 translocation

As discussed above, nucleoplasmic translocation of NPM1 is the most prominent hallmark of nucleolar stress. However, the upstream causes of this translocation remained unclear. In other words, how various cellular insults trigger NPM1 translocation had not been ever asked.

**Figure 4:**
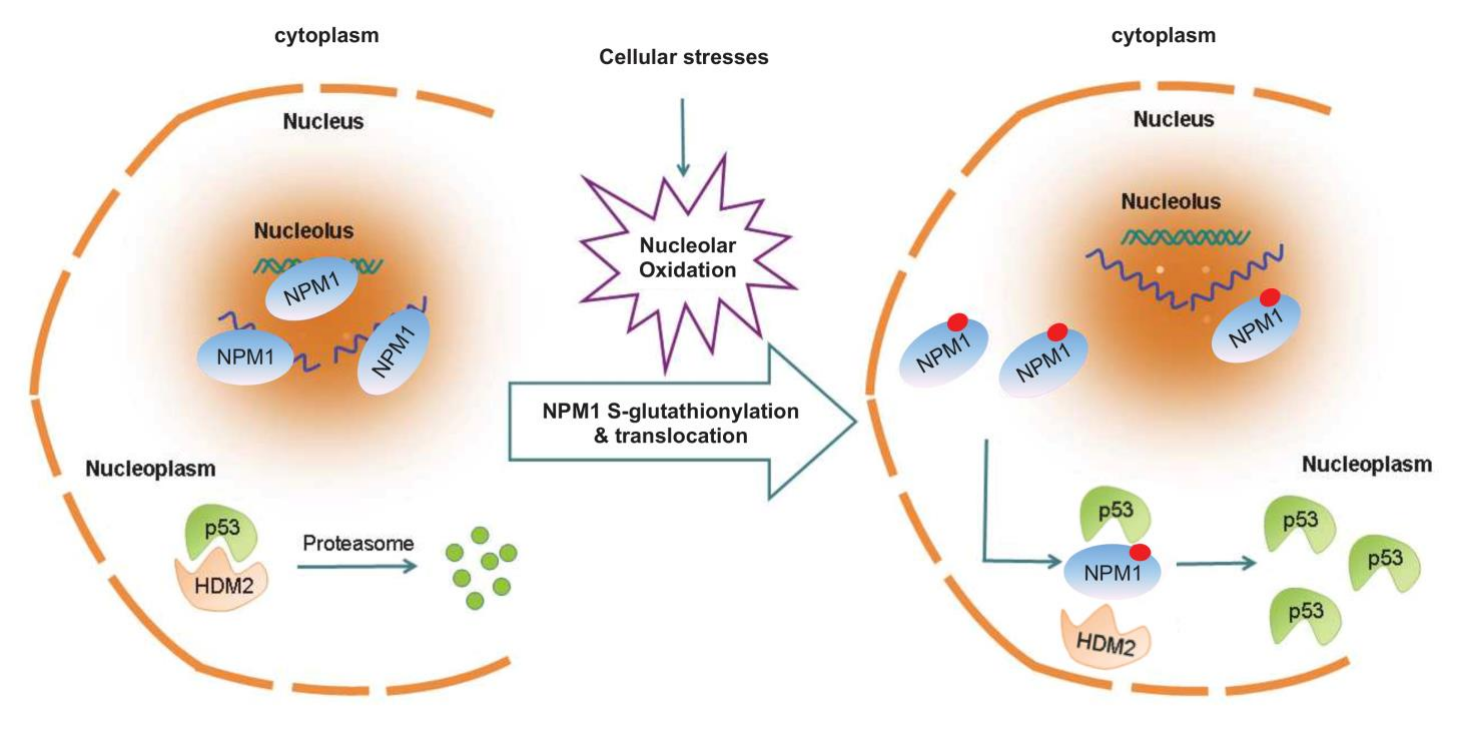
FIGURE 4: NPM1 sensing for nucleolar stress. Nucleolar oxidation is a general response to nucleolar stress. S-glutathionylation and nucleoplasmic translocation of NPM1 are indispensable for p53 activation in nucleolar stress 31.

Using single live-cell imaging and the redox biosensors, we demonstrated that nucleolar oxidation is a general response to various cellular stresses and a trigger for NPM1 translocation. This conclusion was supported by that antioxidant N-acetyl-cystein pretreatment was able to prevent the NPM1 translocation to a great extent, while treatment with a protein reducing agent completely inhibited NPM1 translocation. We showed that during nucleolar oxidation, NPM1 undergoes S-glutathionylation on cysteine 275, which triggers the dissociation of NPM1 from nucleolar nucleic acids. Accordingly, the NPM1 C275S mutant, unable to be glutathionylated, remained in the nucleolus under nucleolar stress, and greatly compromised the activation of p53. In sum, our findings provide a redox mechanism underlying the nucleolar stress sensing by NPM1 31 (**Figure 4**).

## NUCLEOLAR STRESS AND HUMAN DISEASES

Natural mutations in genes that encode ribosomal proteins or proteins regulating ribosome biogenesis within the nucleolus result in a class of genetic disorders entitled ‘ribosomopathies’. These diseases usually have dramatic systemic phenotypes and severe outcomes 137,138,139,140,141,142,143,144. Of note, although ribosomopathies display nucleolar stress, there are no therapeutic options directly targeting nucleolar stress to delay disease progression.

Nucleolar stress is a common event in neurodegenerative disorders such as Parkinson's disease (PD) 137, Alzheimer's disease (AD) 80 and Huntington's disease (HD) 141. Despite the fact that these disorders are polycausal, nucleolar stress may be one of the significant mediators in the degeneration or loss of neurons 138. Besides morphological and functional manifestations in tissues and cell culture, the direct causal relation between these diseases and nucleolar stress has been established using the TIF-IA mouse model (see below) 79,145. A related application, aiming to alleviate nucleolar stress for the prevention and treatment of these disorders will likely ensue.

Large and abnormal nucleoli are commonly observed in cancer cells 146. The hyperactivation of ribosome biogenesis likely contributes to increased cancer cell survival and proliferation. In addition, cancer treatment faces challenges in chemo-radio-resistance cancers and those insensitive to other killing approaches. Potentiating nucleolar stress in these cancer cells may be a novel therapeutic strategy. Indeed, some typical nucleolar stress-inducing agents are under clinical investigation for remedy of leukemia, which has shown promising outcomes 147,148.

Hereafter, we present a brief overview on these two types of nucleolar stress-related diseases. We recommend several comprehensive and in-depth reviews 137,147,149,150, and try to provide some updated information.

### Neurodegenerative disorders

Neurodegenerative disorders are chronic diseases, characterized by the progressive loss of specific neurons in the central or peripheral nervous system. These diseases are characterized by degeneration or loss of a specific subpopulation of neurons. Nucleolar stress is an emerging element of the degenerative process, caused by impaired rRNA transcription and altered nucleolar integrity 151.

PD is associated with the loss of dopaminergic (DA) neurons. Reduced nucleolar volume usually reflects reduced rRNA synthesis, while reduced rRNA synthesis has been reported in neurodegenerative disorders 152. Early data show that nucleolar volume in DA neurons is decreased in PD patients and this is inversely correlated with disease duration 153,154. Decreased nucleolar volume has been reported in the partial unilateral intrastriatal 6-hydroxydopamine oxidative stress rat model of PD 81. Along these lines, a pharmacological mouse model of PD displays disruption of nucleolar integrity 79, while there is also a significant atrophy of the nucleoli in AD 80. Interestingly, DNA damage in neurons can cause NPM1 translocation to the nucleoplasm 155, thus eliciting another hallmark of nucleolar stress.

Mouse models for conditional knockout (KO) of the transcription factor TIF-IA have been generated, in which nucleolar stress is induced in specific neuronal populations at a defined time-point 44. Therefore, mice with conditional KO of TIF-IA not only confirmed a causal correlation of nucleolar stress with neuronal degeneration, but also served as an efficient model to study nucleolar stress itself. Cell-specific TIF-IA KO in distinct postmitotic neurons resulted in their slow, progressive degeneration, showing that neurons can survive for several months under nucleolar stress 79,145,156,157. This slow progression allows the analysis of the sequence of events triggered by nucleolar stress in distinct neuronal populations. Interestingly, DA-specific TIF-IA KO mice show behavioral and cellular features of PD 79. Notably, nucleolar impairment at the age of two months in DA neurons leads to mitochondrial dysfunction and increased oxidative damage, characteristics shared by various neurodegenerative diseases, such as PD, AD and HD. Interestingly, DA-specific TIF-IA KO in a double KO background of the PD-related genes DJ-1/PINK1 displayed an early phenotype similar to those mice lacking TIF-IA alone 158. This conditional TIF-IA KO mouse model has also revealed a dual role of nucleolar stress. It could trigger a neuroprotective defense response at early stages, probably through inducing autophagy 145 and p53-dependent antioxidant response 159, but, on the long run, lead to impaired mitochondrial function and increased oxidative stress, and ultimately neuronal death 79,145.

Further dissection of the regulatory factors in nucleolar stress at a temporal resolution may help to better understand the pathophysiology of neurodegenerative disorders and create novel interfering strategies for prevention and treatment.

### Malignancies

Given the pivotal role of NPM1 in nucleolar stress transmission to p53, one of the main tumorsuppressors, there are multiple NPM1-based therapeutic strategies for cancer treatment. Indeed, a vast part of anticancer drugs triggers apoptotic cell death through p53-activation pathway. Because of a causal relationship between anticancer chemical drug induced cell apoptosis and NPM1 translocation 63,134,160, NPM1 translocation could be regarded as a effective drug screening marker for novel antitumor agents selection 8. As a safe and tolerable drug in clinical phase II trial, CIGB-300 exerts a broad original and synergistic antiproliferative effect on different cell lines 161,162,163,164. Perera et al. revealed that CIGB-300 directly binds to NPM1, induces a rapid nucleoplasmic translocation of NPM1, and leads to a nucleolar disassembly-dependent apoptosis 165.

Additionally, there are also some selective Pol I inhibitors that reveal promising clinical effects 148,166. For example, CX-3543 exhibits broad anti-proliferative and apoptotic effects on cancer cells and demonstrated impressive anti-tumor growth properties in xenograft models of breast and pancreatic cancer 167. Intriguingly, the next generation of CX-3543, CX-5461 showed effectiveness in human cancer cells that experience overloaded ribosomal biogenesis compared to normal cells 168,169.

The cytotoxic marine natural product Avrainvillamide specifically binds the C-terminus of NPM1 and leads to its disassociation from nucleolar nucleic acid 170,171. Furthermore, a G-quadruplex ligand TmPyP4 could perfectly compete and inhibit nucleolar nucleic acid binding activity of NPM1 172. Both of them caused NPM1 nucleoplasmic translocation. Because the NPM1 C-terminal domain surface is responsible for NPM1 nucleolar localization 173, it is likely that these two C-terminus targeting compounds are also related with nucleolar stress.

Additionally, there are some compounds targeting other NPM1 functional domains. NSC348884 and YTR107 interact with the N-terminus of NPM1 and inhibit its normal oligomerization function 174. REV-NLS targets the N-terminal NPM1 surface and inhibits its protein-protein interaction functions 175. A small synthetic RNA, named 1A1, identified in a screen for binding to full-length NPM1, binds to the central region of NPM1 176. These NPM1-targeting compounds both showed excellent anticancer effects. Importantly, both of them induced increased p53 levels and trancriptional activity, although their effects on NPM1 localization has not been investigated so far.

The* NPM1* gene was identified to harbor the most frequent genetic (30%) lesions in adult acute myeloid leukemia (AML) 177,178. Immunohistochemical staining in bone marrow specimens reveals a constant cytoplasmic localization for NPM1 179. Due to a frame shift mutation in one allele of the last exon of *NPM1* gene 180, this mutant, termed as NPMc+ (cytoplasmic positive), holds a nuclear export signal (NES) and a misfolded structure in its C-terminus, resulting in disassociation from nucleic acid and cytoplasmic export 173,181,182. In addition, this mutant also causes a cytoplasmic retention effect of wild-type NPM1, leaving only trace amounts of wild-type NPM1 in the nucleoli 183. Fortunately, an intact functional p53 response pathway is still preserved in NPM1c+ AML cells 178, thus NPM1c+ AML cells remain sensitive to nucleolar stress-induced p53 activation 184,185. Thus, Falini *et al*. focused on Act.D induced nucleolar stress and utilized the clinical recommended dose of Act.D to treat seven refractory or relapsed NPM1c+ mutant only (without FLT3 mutations) AML patients. Three of them showed hematologic complete remission within six weeks of therapy, and one of them even manifested molecular complete remission lasting for 14 months 186.

Considering our recently uncovered sensing mechanism of NPM1 in response to nucleolar stress 31, we strongly expect that the chemical compounds that enable NPM1 glutathionylation or specifically target the NPM1 nucleic acid binding site would exert better efficacy in therapies for malignancies like NPM1c+ AML.

## Abbreviations

Act.D: Actinomycin D
AD: Alzheimer’s disease
AML: acute myeloid leukemia
ARF: alternative reading frame
CBS: cystathionine-β-synthase
DFC: dense FC
FC: fibrillar center
GC: granular component
KO: knockout
PD: Parkinson’s disease
Pol I: RNA Polymerase I
rDNA: ribosomal DNA
RP: ribosomal protein
rRNA: ribosomal RNA
TF: transcription factor
